# Long COVID Clinical Severity Types Based on Symptoms and Functional Disability: A Longitudinal Evaluation

**DOI:** 10.3390/jcm13071908

**Published:** 2024-03-26

**Authors:** Manoj Sivan, Adam B. Smith, Thomas Osborne, Madeline Goodwin, Román Rocha Lawrence, Sareeta Baley, Paul Williams, Cassie Lee, Helen Davies, Kumaran Balasundaram, Darren C. Greenwood

**Affiliations:** 1 Leeds Institute of Rheumatic and Musculoskeletal Medicine, University of Leeds, Leeds LS7 4SA, UK; t.osborne@leeds.ac.uk (T.O.); m.e.l.goodwin@leeds.ac.uk (M.G.); r.a.rochalawrence@leeds.ac.uk (R.R.L.); 2 COVID Rehabilitation Service, Leeds Community Healthcare NHS Trust, Leeds LS11 0DL, UK; 3 National Demonstration Centre of Rehabilitation Medicine, Leeds Teaching Hospitals NHS Trust, Leeds LS7 4SA, UK; 4 School of Medicine, University of Leeds, Leeds LS2 9JT, UK; a.b.smith@leeds.ac.uk (A.B.S.); d.c.greenwood@leeds.ac.uk (D.C.G.); 5 Leeds Institute for Data Analytics, University of Leeds, Leeds LS2 9JT, UK; 6 ELAROS 24/7 Digital Company, Sheffield S1 2BJ, UK; 7 Birmingham Community Healthcare NHS Trust, Birmingham B7 4BN, UK; s.baley@wlv.ac.uk; 8 Hertfordshire Community NHS Trust, Welwyn AL6 9PW, UK; paul.williams80@nhs.net; 9 Imperial College Healthcare NHS Trust, London W2 1NY, UK; cassie.lee1@nhs.net; 10 Cardiff and Vale University Hospitals NHS Trust, Cardiff CF14 4XW, UK; helen.davies30@wales.nhs.uk; 11 NIHR Leicester Biomedical Research Centre—Respiratory & Infection Theme, Glenfield Hospital, Leicester LE3 9QP, UK; kumaran.balasundaram@uhl-tr.nhs.uk

**Keywords:** SARS-CoV-2, post-COVID-19 condition, post-COVID-19 syndrome, C19-YRS, phenotypes, LOCOMOTION

## Abstract

**Background**: Long COVID (LC) is a multisystem clinical syndrome with functional disability and compromised overall health. Information on LC clinical severity types is emerging in cross-sectional studies. This study explored the pattern and consistency of long COVID (LC) clinical severity types over time in a prospective sample. **Methods**: Participants with LC completed the condition-specific outcome measure C19-YRSm (Yorkshire Rehabilitation Scale modified version) at two assessment time points. A cluster analysis for clinical severity types was undertaken at both time points using the k-means partition method. **Results**: The study included cross-sectional data for 759 patients with a mean age of 46.8 years (SD = 12.7), 69.4% females, and a duration of symptoms of 360 days (IQR 217 to 703 days). The cluster analysis at first assessment revealed three distinct clinical severity type clusters: mild (*n* = 96), moderate (*n* = 422), and severe (*n* = 241). Longitudinal data on 356 patients revealed that the pattern of three clinical severity types remained consistent over time between the two assessments, with 51% of patients switching clinical severity types between the assessments. **Conclusions**: This study is the first of its kind to demonstrate that the pattern of three clinical severity types is consistent over time, with patients also switching between severity types, indicating the fluctuating nature of LC.

## 1. Introduction

Long COVID (LC) involves a potential plethora of persistent symptoms which continue to be experienced by some patients four weeks after a confirmed or possible COVID-19 infection [1]. The term encompasses the National Institute for Health and Care Excellence (NICE)-defined terms, ‘ongoing symptomatic COVID-19′ (persistent symptoms 4–12 weeks after the infection) and ‘Post-COVID Syndrome (PCS)’ (symptoms persisting beyond 12 weeks after infection that are not attributable to alternative diagnosis) [2]. The World Health Organisation (WHO) prefers the term post-COVID condition (PCC) for patients experiencing continued symptoms three months after a confirmed or possible infection that lasts at least two months [3]. There are an estimated 1.9 million cases of LC in the UK alone and more than 200 million cases worldwide [4,5]. LC is a multisystem syndrome with more than 200 symptoms reported across 10 organ systems with common symptoms being fatigue, pain, breathlessness, brain fog (cognitive problems), sleep problems, palpitations, dizziness, anxiety, depression, post-traumatic stress, skin rash, and allergic reactions [6]. It is a fluctuating condition with a protracted course causing significant distress and disability to the individual [4].

The clinical symptoms and impact of LC have been recorded using a variety of generic and condition-specific patient reported outcome measures (PROMs). The C19-YRS (COVID-19 Yorkshire Rehabilitation Scale) was the literature’s first published and validated PROM for LC [7], and it has been recommended by both NICE and NHS England [8]. The scale has been used in several LC studies which have indicated support for the content and construct validity, as well as the responsiveness of the scale to reliably capture persistent symptoms [9,10,11,12]. C19-YRS gives scores in three domains: symptom severity (SS), functional disability (FD), and overall health (OH). There are other condition-specific and generic instruments also being used to characterize LC [13,14,15].

Clinical severity types or clinical severity phenotypes for LC have been described in the literature [16,17,18,19,20]. The post-hospitalisation COVID-19 study (PHOSP-COVID), which involved 1077 patients, identified four clusters of post-hospitalisation LC patients with varying severities of mental and physical health impairment, described as mild, moderate, severe, and very severe, with participants typically reporting nine persistent symptoms five months after discharge [16]. Our previous cross-sectional study of 370 predominantly non-hospitalised patients completing the C19-YRS scale showed three distinct clinical severity phenotypes in LC: mild, moderate, and severe [17].

The consistency of the aforementioned clinical severity types over time and the relationship between SS, FD, and OH in the context of the severity types has so far not been explored longitudinally. The aim of this study was to understand how the clinical severity types fluctuate in a community LC cohort at two assessment points and whether there is a linear relationship between the three domains of the health condition (SS, FD, and OH) longitudinally.

## 2. Materials and Methods

### 2.1. Setting

This prospective study is part of the NIHR-funded Long COVID Multidisciplinary Consortium Optimising Treatments and Services Across the NHS (LOCOMOTION) mixed-methods study involving ten LC services across the UK. Ethics approval for the LOCOMOTION study was obtained from the Bradford and Leeds Research Ethics Committee on behalf of the Health Research Authority and Health and Care Research Wales (reference: 21/YH/0276) on 6 January 2022 [21].

### 2.2. Participants

Participants were included in the study if they met the following criteria:

- They had a diagnosis of LC. They were not required to have had a positive polymerase chain reaction (PCR) test or antibody test for SARS-CoV-2, as per NICE definition [2]. These tests were not widely available to the general population of the UK at the start of the pandemic.
- They were receiving management for the condition from a LOCOMOTION study participating LC service.
- They had LC symptoms which could not be explained through alternative medical diagnosis.
- They were registered on the ELAROS digital PROMs platform [22] and were required to complete PROMs every three months after being registered.

Participants were recruited consecutively and agreed to their data being used anonymously for research purposes or service evaluation. Participants were asked to complete the modified version of C19-YRS (C19-YRSm) every three months after being registered.

### 2.3. C19-YRS Instrument

The C19-YRSm is a 17-item instrument designed to capture the key symptoms of LC as well as its impact on activities of daily living and overall health [7]. The SS subscale captures the severity of symptoms including breathlessness, cough, fatigue, altered smell/taste, pain or discomfort, cognitive problems, mood, palpitations, post-exertional malaise (PEM) or post-exertional symptom exacerbation (PESE), and sleep problems. The FD subscale focusses on the impact of LC on daily functions: communication, mobility, personal care, activities of daily living, and social functioning. Responses in SS and FD subscales are rated on a 0 (no symptom or dysfunction) to 3 (severe life-disturbing symptom or dysfunction) Likert scale. In addition, there is an OH subscale scored on a 0–10 Likert scale, with 0 representing worst health and 10 representing best health. Finally, patients are also asked to indicate whether they have experienced other symptoms (OS) over the last seven days (including any that have worsened following their illness). The OS list comprises 25 symptoms (including, fever, skin/rash/discolouration of skin, allergy, and hair loss), as well as a free text option to include additional symptoms that are not on the list. The items of the scale span all aspects of the 2001 WHO International Classification of Functioning, Disability, and Health (ICF) framework [23].

### 2.4. Statistical Analysis

All analyses were performed using R Studio (R version 4.1.1). Patient demographic and clinical information were summarised using descriptive statistics (count and percentage).

Inferential statistics were used to analyse the C19-YRSm data across two timepoints (Assessments A1 and A2). The SS, FD, and OH subscale scores were summarised using means and standard deviations. The OS subscale was summarised using frequency data (count and percentages). The degree of association between symptoms and functional items was evaluated using the Spearman correlation and represented in a heat map. Symptoms were categorised as severe (>2) or not severe (0 to 2). A cluster analysis for clinical severity types was undertaken using the k-mean partitioning method using two, three, and four initial clusters and different starting values. The clinical severity type structure was assessed at A1 and A2 time points. The patient clinical severity type allocation was evaluated with Kendall’s tau and (unweighted) kappa to assess the level of intra-patient association and agreement, respectively, to determine whether patients remained in the same severity type cluster over time. In addition to this, the data at A2 were split by the median number of days (duration between assessments) to determine whether any cluster structure identified was independent of the time interval between assessments.

Polychoric (confirmatory) factor analyses (no rotation) were undertaken on the SS and FD subscales to evaluate the underlying factor structures (i.e., the presence or otherwise of symptom-type clusters).

### 2.5. Role of the Funding Source

The funders of the study had no role in the study’s design, data collection, data analysis, and data interpretation, or in the writing of the report.

## 3. Results

### 3.1. Patients

A total of 759 patients (69.4% females) completed the C19-YRSm at A1 and 356 (68.0% females) patients completed the instrument at both timepoints A1 and A2. The mean time difference between A1 and A2 was 16.2 days (SD = 17.6 days). Demographic details and medical histories at A1 are shown in Table 1.

Most of the patients were Caucasians (*n* = 565, 74%), with 10.4% of the sample being from Black, Asian, or minority ethnic (BAME) groups. The mean age was 46.8 years (SD = 12.7 years). The mean weight was 82 kg (SD = 23 kg), and the mean body mass index (BMI) was 28 kg/m^2^ (SD = 8.4 kg/m^2^). Just over half (*n* = 405, 53.4%) had never smoked. Over a fifth (*n* = 177, 23%) of patients had no job changes resulting from COVID-19; on the other hand, 14% (*n* = 107) and 19% (*n* = 146) were on reduced working hours or on sick leave due to LC, respectively. A further 22% (*n* = 167) had to make changes to their current employment due to LC.

The mean SS score was 17.5 (SD = 5.71), the mean FD score was 7.0 (SD = 3.81), and the OH mean score was 4.7 (SD = 1.89).

### 3.2. Cluster Analysis

**Figure 1:**
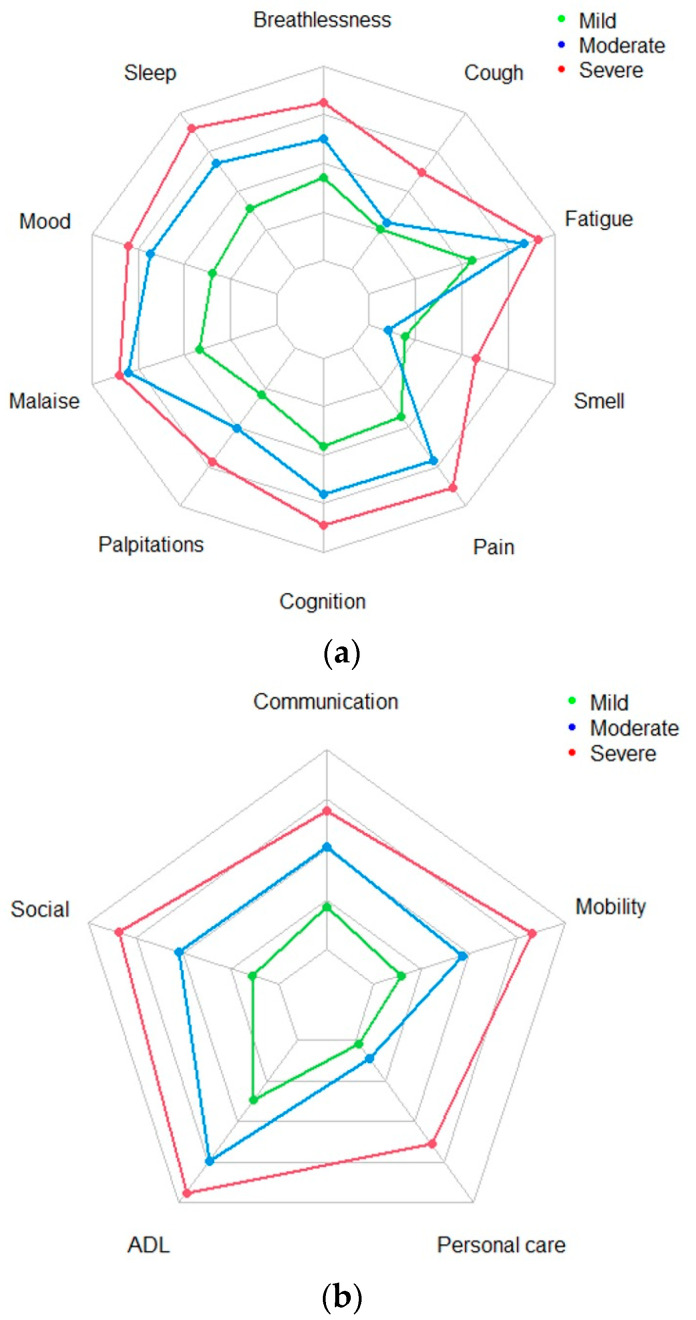
(**a**) Radar plot for symptom severity—Assessment 1. (**b**) Radar plot for functional disability—Assessment 1.

**Figure 2:**
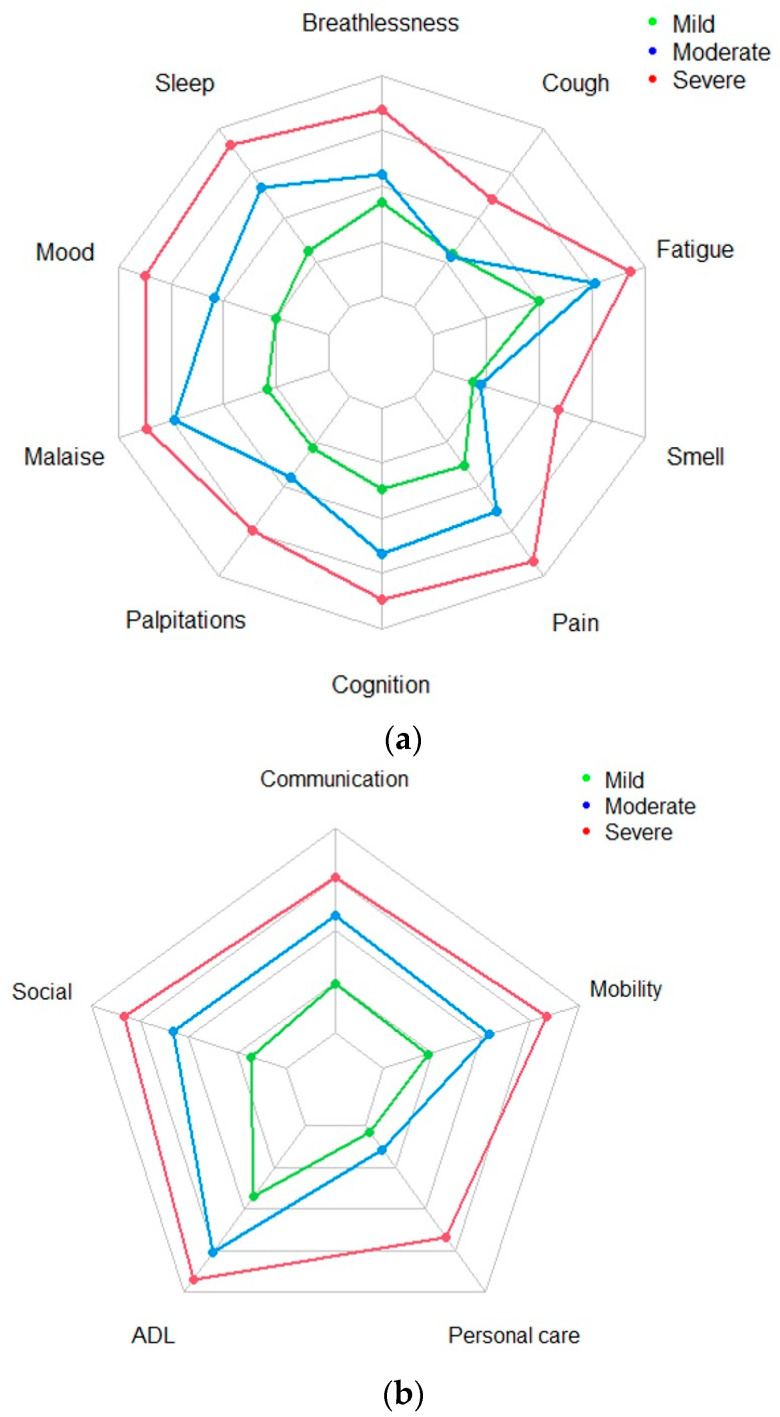
(**a**) Radar plot for symptom severity—Assessment 2. (**b**) Radar plot functional disability—Assessment 2.

The cluster analysis did not reveal any symptom-type clusters but distinct severity-type clusters were observed. The plots are shown in Figure 1a,b for A1 and Figure 2a,b for A2. For both the SS (Figure 1a and Figure 2a) and FD (Figure 1b and Figure 2b) subscales of the C19-YRSm, three distinct clinical severity types were observed. Two groups reflected “mild” and “severe” symptom severity and dysfunction severity. The third group had “moderate” symptom severity, which were indicated by high average scores for fatigue and PEM, low scores for cough and smell, and moderate scores for the remaining symptoms and functions. Patients with a low score on a particular symptom also scored low on the other symptoms. Similarly, patients with more severe symptoms were also, on average, more likely to record severe symptoms on the other symptom items. The FD subscale also had a similar three severity-type pattern.

The same pattern was also observed at A2 (Figure 2a,b), including for the median time split (Supplementary Figure S1A,B), indicating that the clusters of severity types for SS and FD were consistent over time and independent of the time interval between assessments.

In terms of the relationship between SS and FD, the absolute agreement between severity-type clusters for SS and FD at A1 was moderate at 59%, with a Kendall’s tau of 0.54 (Table 2), suggesting that around 41% of patients had different severity-type clusters of SS and FD, with a kappa of 0.38 (95% confidence interval (CI) = 0.35 to 0.40). The same pattern was observed at A2 (for those patients who had completed the C19-YRSm at two assessments, *n* = 336): absolute agreement 0.60, Kendall’s tau 0.59, and kappa 0.4 (95% CI: 0.36 to 0.44), as shown in Table 3.

Table 4 and Table 5 show the proportion of patients by severity-type cluster for SS and FD separately at A1 and A2, i.e., change in severity by the two cluster types over time. For SS (Table 4), 49% of patients fell into the same three categories at the two assessment times (e.g., “mild severity type” at A1 and A2). Conversely, therefore, just over half (51%) fell into different severity-type cluster categories at the two timepoints. Kendall’s tau was 0.33 and kappa was 0.23 (95%CI = 0.19 to 0.27). Similarly, for FD (Table 5), 47% of patients remained in the same severity category at the two assessment times; Kendall’s tau was 0.25 and kappa 0.19 (95%CI: 0.15 to 0.24).

Overall health was categorized as “poor” (0 to 2), “moderate” (3 to 6), or “good” (7 to 10). Absolute agreement in these three OH categories between A1 and 2 was 65.4%; Kendall’s tau was 0.22 and kappa was 0.25 (95%CI: 0.15 to 0.34). This suggests that, although OH was more stable (than SS and FD) over time for the majority of patients, around a third of patients were experiencing changes in their OH status (Table 6).

**Figure 3:**
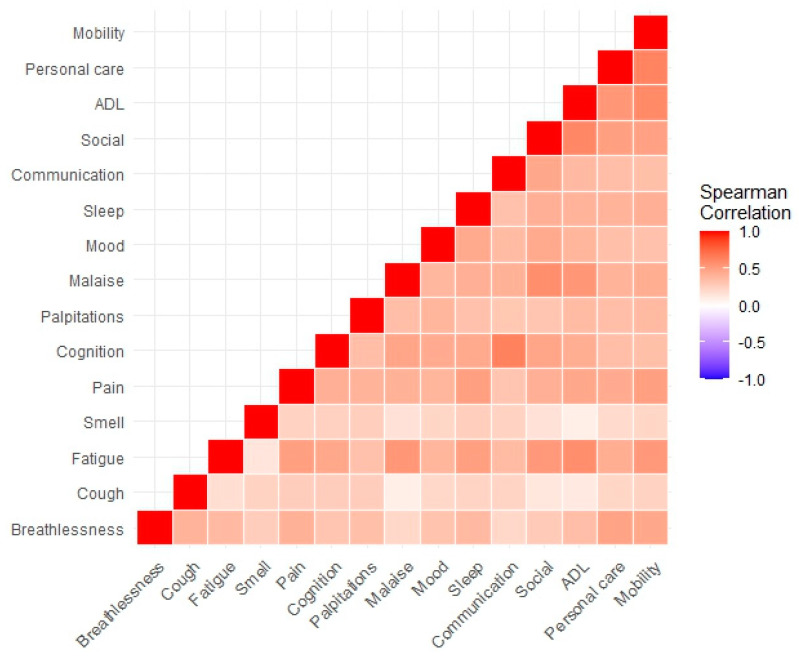
Heat map showing the spearman correlation values between symptoms and functional disability items.

The results of the Spearman correlation analysis showed no distinct groupings of symptoms (rho range = 0.09 to 0.64), but, in general, moderate-to-good levels of association between the individual symptoms and FD items (Figure 3). This validates the construct of the C19-YRS instrument and the need to include all the items to capture LC.

### 3.3. Polychoric Factor Analysis (PFA)

The results of the PFA for both the SS and FD subscales revealed a single underlying factor explaining 41% and 45% of the variance for the A1 and A2 data, respectively, (eigen values 4.1 and 4.5) for the SS subscale, and 60% and 62% of the variance for the FD subscale (A1 and A2, respectively). These results indicate a unidimensional structure for the C19-YRSm SS and FD subscales (Supplementary Tables S1 and S2; Supplementary Figures S2 and S3).

## 4. Discussion

This is the first longitudinal study of its kind in the current literature to show the consistency of clinical severity types in LC. The three broad severity-type clusters identified were mild, moderate, and severe, with this structure pattern seen for both SS and FD aspects of the condition. The severity-type three-cluster structure is consistent over time with a significant number of participants in the study (51%) switching between cluster types, highlighting the fluctuating nature of the condition. The fluctuation is independent of the severity of the condition and the time between the assessments.

The existence of severity-type clusters and the absence of symptom types in this cohort highlights the common underlying mechanisms of LC symptoms. The current literature suggests that some of the possible mechanisms are immune activation, autoantibodies, dysautonomia, endothelial damage (hypercoagulability), immune dysregulation, and viral persistence [24,25]. The lack of clear individual symptom-type clusters in this study supports the assumption that there is an overlap of these mechanisms and the possible presence of more than one mechanism in relation to the host response to the viral infection. The sample of participants in this study represents a cohort from specialist LC services, with most of them having a prolonged duration of symptoms [average mean duration at A1 was 408.8 days (SD = 260.7 days); median was 323 days (interquartile range: 203.0 to 562.0 days)]. These participants are more likely to have complex multiple mechanisms attributed to the persistence and fluctuation of symptoms.

The findings of clinical severity types and the fluctuating nature of the condition have widespread implications for self-management and the interventions provided by healthcare professionals, as well as the support provided by carers and family members. There is established evidence for PEM or PESE in the literature as a hallmark finding in LC and other post-viral illness [26,27,28]. Relapse can be triggered by physical, cognitive, or emotional exertion, and evidence suggests that cognitive and emotional exertion can be responsible in equal measure to physical exertion. Studies also suggest that fatigue and PEM are the symptoms that are most frequently experienced as severe (and occur most frequently with other severe symptoms) [17].

The stratification based on clinical severity could help national and local providers to plan services and interventions that might be directed towards these categories at the time of presentation. Mild severity can be monitored in primary care services (such as general practitioners) and offered resources such as the ‘Your COVID Recovery’ website [29], WHO self-management booklet [30], or the ELAROS self-management platform [22] which offers a range of resources from English LC services. The moderate and severe cases and those with a significant FD can be offered specialist input in dedicated LC services by specialist multidisciplinary team (MDT) staff. However, it needs to be highlighted that the condition is fluctuating in nature and clinical pathways need to allow flexibility to change management plans based on severity of the condition.

There is some literature suggesting symptoms such as fatigue or anxiety or mood disorders to be highly prevalent in the general population prior to infection [31,32]. We were, however, able to show in this study that, for many participants, these were not pre-existing prior to infection, albeit scored retrospectively. This finding supports the de novo (new onset) nature of the symptoms attributable to LC. The data collected in this study also suggest that C19-YRSm can be used to capture pre-illness symptoms, even though there is likely to be a certain degree of recall bias.

This study highlights the importance of using condition-specific outcome measures, as such an analysis would be almost impossible to undertake using a combination of generic measures (such as separate measures for each LC symptom). The factor analysis carried out indicates that there is likely to be a single underlying factor (i.e., LC) for the SS and FD domains. The modest correlation supports the conceptual design of having separate SS and FD subscales within the C19-YRSm PROM. The OH subscale was shown to be less fluctuating when compared to SS and FD and can be used to estimate the overall condition trajectory and its impact on well-being. The non-linear relationship between SS, FD, and OH is in keeping with the WHO ICF framework for any health condition [23].

Our study findings are helpful for both individuals with LC and clinicians managing the condition. Individuals with LC are well aware that the condition is fluctuant, and they experience a relapse (with worsening of severity of symptoms) with particular triggers (physical, cognitive, or emotional exertion). Understanding this model helps them adapt their lifestyle to avoid frequent relapses and stabilise the condition (and its recovery). Clinicians can categorise the severity types and plan their management accordingly. When symptoms and disability are severe, they may initiate certain treatments (such as medications) to provide symptomatic relief. Clinicians must conceptualise the condition as multisystemic with common underlying mechanisms rather than isolated symptoms related to one organ system.

This study has several limitations. The first is choosing the parameters for the cluster analysis, as applying a three-cluster structure results in a three-cluster structure. However, the cluster structure holds when the data are interrogated in different ways, i.e., this does reflect a stable three-cluster clinical severity type structure. Also, symptom-type clusters could not be identified despite trying various possible options for analysis. Second, our sample may not be representative of LC generally in the community. The sample comprised predominantly patients with LC for at least six months and symptoms which are severe enough to be referred to specialist LC clinics. It is possible that milder cases not presenting to LC clinics for management might report symptoms in a different manner. Also, confirmatory PCR tests were not required for a clinical diagnosis of LC. This, however, does not invalidate the results, as the diagnosis of LC is still a clinical diagnosis and does not depend on a confirmatory PCR test. There was a predominance of white female patients, which highlights the possibility of health inequalities whereby many patients from black and minority ethnic backgrounds had not received a referral to the participating LC clinics. We did not explore the influence of comorbidities and vaccination status on severity types. Another limitation is that we can only investigate the consistency of severity-type patterns in those with repeated measures, as 53% of registered patients did not complete a second assessment. This, however, does not refute the finding of consistent patterns in those who completed the second assessment and is in keeping with other studies that have reported on fluctuations seen in the condition [33,34]. Finally, it is worth noting that symptoms and their severity were self-reported by participants, so there could be a degree of subjectivity in their recording, in that they tend to grade severity similarly across the symptoms. This limitation. however, applies to all subjectively reported patient-reported outcome measures.

## 5. Conclusions

This is the first study to report on the consistency of LC clinical severity types over time in a prospective longitudinal study. Clinical severity types might help understand the fluctuating nature of LC and improve the ability to stratify patients for targeted interventions and the planning of care pathways. This study highlights the advantage of using a condition-specific outcome measure which has separate subscales for SS, FD, and OH, which are not necessarily linearly related. Further research is needed to understand the relationship between condition fluctuations and long-term condition trajectories in LC.

## Figures and Tables

**Table 1:** Demographics at Assessment 1 and symptom severity scores.

	All (*n* = 759)	Mild (*n* = 96)	Moderate (*n* = 422)	Severe (*n* = 241)
Female	527 (69.4%)	69 (71.9%)	291 (69.0%)	167 (69.3%)
Mean age (SD) in years	46.8 (12.7)	46.2 (11.9)	46.6 (12.7)	47.3 (13.0)
Mean weight (kg) (SD) (*n* = 127)	82.4 (23.0)	79.5 (16.9)	82.0 (22.1)	86.4 (36.1)
Mean BMI (kg/m^2^) (SD) (*n* = 127)	28.0 (8.37)	26.2 (3.95)	28.1 (6.51)	28.8 (10.00)
Ethnicity				
*White (n = 565)*	74.4%	9.0%	60.4%	30.6%
*Black, African, Black British or Caribbean (n = 20)*	2.6%	15.0%	50.0%	35.0%
*Asian (any Asian background) (n = 45)*	5.9%	17.8%	53.3%	28.9%
*Mixed or multiple ethnic groups (n = 14)*	1.8%	41.3%	50.0%	35.7%
*Other ethnicity (n = 11)*	1.5%	27.3%	54.5%	18.2%
*Not recorded (n = 104)*	13.7%			
Smoking status				
*Never smoked (n = 405)*	53.4%	12.8%	55.8%	31.4%
*Current regular smoker (n = 29)*	3.8%	13.8%	65.5%	20.7%
*Current occasional smoker (n = 28)*	3.7%	7.1%	75.0%	17.9%
*Ex-smoker (n = 185)*	24.4%	5.4%	61.6%	33.0%
*Not recorded (n = 112)*	14.8%			
Change in employment status				
*No change (n = 177)*	23.3%	6.8%	62.1%	31.1%
*Lost job (n = 29)*	3.8%	10.3%	51.7%	37.9%
*On reduced working hours (n = 107)*	14.1%	12.1%	60.7%	27.1%
*On sick leave (n = 146)*	19.2%	8.9%	58.2%	32.9%
*Had to retire or change job (n = 35)*	4.6%	5.7%	62.9%	31.4%
*Changes made to role or working arrangements (n = 167)*	22.0%	15.0%	47.3%	37.7%
*Not recorded (n = 98)*	12.9%			
Other details				
Positive COVID-19 test	30.8%			
Mean days admitted to hospital (*n* = 78) (SD)	12.9 (20.3)	10.2 (10.9)	12.3 (20.5)	15.0 (22.3)
Mean symptom severity score at Assessment 1 (SD)	17.5 (5.71)	7.4 (1.84)	15.8 (2.95)	24.0 (2.33)
Mean functional disability score at Assessment 1 (SD)	7.0 (3.81)	2.4 (1.81)	6.0 (3.04)	10.2 (3.02)
Mean overall health score at Assessment 1 (SD)	4.7 (1.89)	6.4 (1.72)	4.9 (1.75)	3.74(1.67)
Mean duration of symptoms (days) (SD)	408.8 (260.7)	390.0 (233.6)	381.9 (243.0)	467.4 (292.9)
Mean interval between Assessments 1 and 2 (days) (SD)	16.2 (17.6)	17.8 (16.9)	16.6 (17.3)	14.8 (18.4)
Median interval between Assessments 1 and 2 (days) (range, min to max)	12.5 (0 to 97)	15.8 (0 to 84)	13.8 (0 to 97)	9.7 (0 to 94)

Symptom severity scores: mild = 1–9, moderate = 10–20, severe = 21–30; BMI = body mass index.

**Table 2:** Proportion of patients in symptom severity and functional disability cluster types: Assessment 1.

	A1 Functional Disability Cluster		
A1 Symptom Severity Cluster	Mild	Moderate	Severe
Mild	0.22	0.10	0.00
Moderate	0.06	0.20	0.11
Severe	0.02	0.11	0.16

Absolute agreement = 0.59; Kendall’s tau = 0.58; kappa = 0.38 (95% confidence interval: 0.35 to 0.40); totals do not sum to one due to rounding; A1 = Assessment 1.

**Table 3:** Proportion of patients in symptom severity and functional disability cluster types: Assessment 2.

	A2 Functional Disability Cluster		
A2 Symptom Severity Cluster	Mild	Moderate	Severe
Mild	0.20	0.02	0.00
Moderate	0.19	0.22	0.04
Severe	0.03	0.12	0.17

Absolute agreement = 0.60; Kendall’s tau = 0.59; kappa = 0.40 (95% confidence interval: 0.36 to 0.44); totals do not sum to one due to rounding; A2 = Assessment 2.

**Table 4:** Proportion of patients switching/remaining in severity cluster types for symptom severity (Assessments 1 to 2).

	A2 Symptom Severity Cluster		
A1 Symptom Severity Cluster	Mild	Medium	Severe
Mild	0.14	0.14	0.05
Moderate	0.05	0.18	0.10
Severe	0.04	0.14	0.16

Absolute agreement 0.49; Kendall’s tau = 0.33; kappa = 0.23 (95% confidence interval: 0.19 to 0.27); A1 = Assessment 1; A2 = Assessment 2.

**Table 5:** Proportion of patients switching/remaining in severity cluster types for functional disability (Assessments 1 to 2).

	A2 Functional Disability Cluster		
A1 Functional Disability Cluster	Mild	Moderate	Severe
Mild	0.17	0.09	0.04
Moderate	0.18	0.18	0.06
Severe	0.07	0.09	0.12

Absolute agreement 0.47; Kendall’s tau = 0.25; kappa = 0.19 (95% confidence interval: 0.15 to 0.24). A1 = Assessment 1; A2 = Assessment 2.

**Table 6:** Proportion of patients switching/remaining in severity types for overall health (Assessments 1 to 2).

	A2 Overall Health Cluster		
A1 Overall Health Cluster	Mild	Moderate	Severe
Mild	0.04	0.05	0.01
Moderate	0.06	0.54	0.11
Severe	0.01	0.10	0.07

Absolute agreement 0.65; Kendall’s tau = 0.25; kappa = 0.25 (95% confidence interval: 0.19 to 0.30); totals do not sum to one due to rounding; A1 = Assessment 1; A2 = Assessment 2.

## Data Availability

The datasets used and analysed during the current study are available from the corresponding author upon reasonable request.

## Supplementary Materials

The following supporting information can be downloaded at: https://www.mdpi.com/article/10.3390/jcm13071908/s1, Figure S1A. Radar plot for symptom severity (median time split); Figure S1B. Radar plot for functional disability (median time split); Figure S2. Factor structure of symptom severity—Assessments 1 and 2; Figure S3. Factor structure of functional disability—Assessments 1 and 2; Table S1. Polychoric factor analysis for symptom severity—Assessments 1 and 2; Table S2. Polychoric factor analysis for functional disability—Assessments 1 and 2.
