# Supplementary material for: Long COVID Clinical Severity Types Based on Symptoms and Functional Disability: A Longitudinal Evaluation
Source: J Clin Med. 2024 Mar 26;13(7):1908. doi: 10.3390/jcm13071908 (PMC11012375; doi:10.3390/jcm13071908)

Supplementary Figure S1A. Radar plot for Symptom Severity (on a subset of 359 patients who had A2 assessment before the median time of 12.5 days)

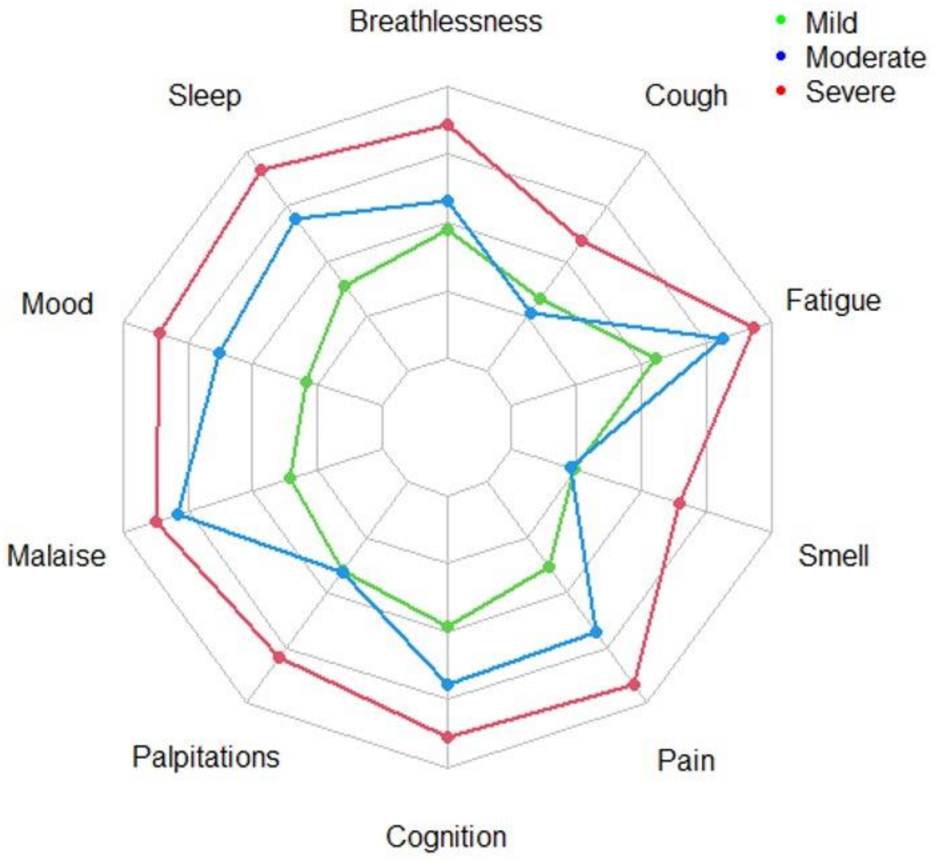

Supplementary Figure S1B. Radar plot for Functional Disability (on a subset of 359 patients who had A2 assessment before the median time of 12.5 days)

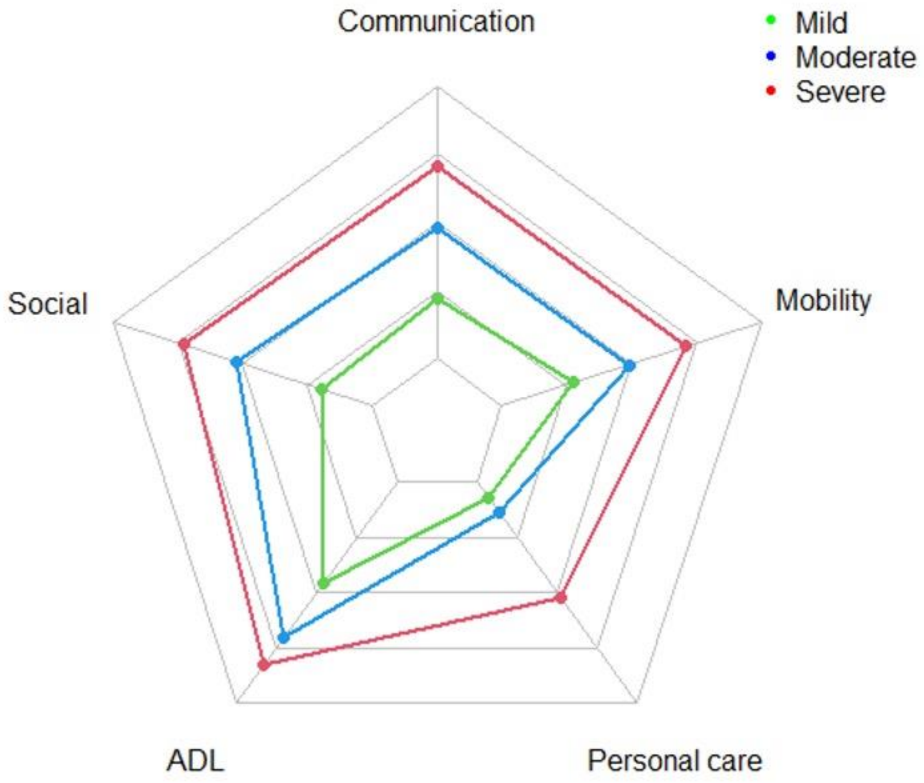

Supplementary Table S1. Polychoric Factor Structure Symptom Severity – Assessments 1 and 2

**Assessment 1 – Factor loadings**		**Assessment 2 – Factor Loadings**	
Breathlessness	0·55	Breathlessness	0·63
Cough	0·40	Cough	0·37
Fatigue	0·77	Fatigue	0·80
Smell	0.41	Smell	0·46
Pain	0·73	Pain	0·73
Cognition	0·72	Cognition	0·79
Palpitations	0·60	Palpitations	0·63
Malaise	0·68	Malaise	0·70
Mood	0.66	Mood	0·71
Sleep	0·74	Sleep	0·76
Eigenvalue	4.10	Eigenvalue	4·50
Proportion Variance explained	0·41	Proportion Variance explained	0·45

Supplementary Figure S2: Factor Structure – Symptom Severity – Assessments 1 and 2

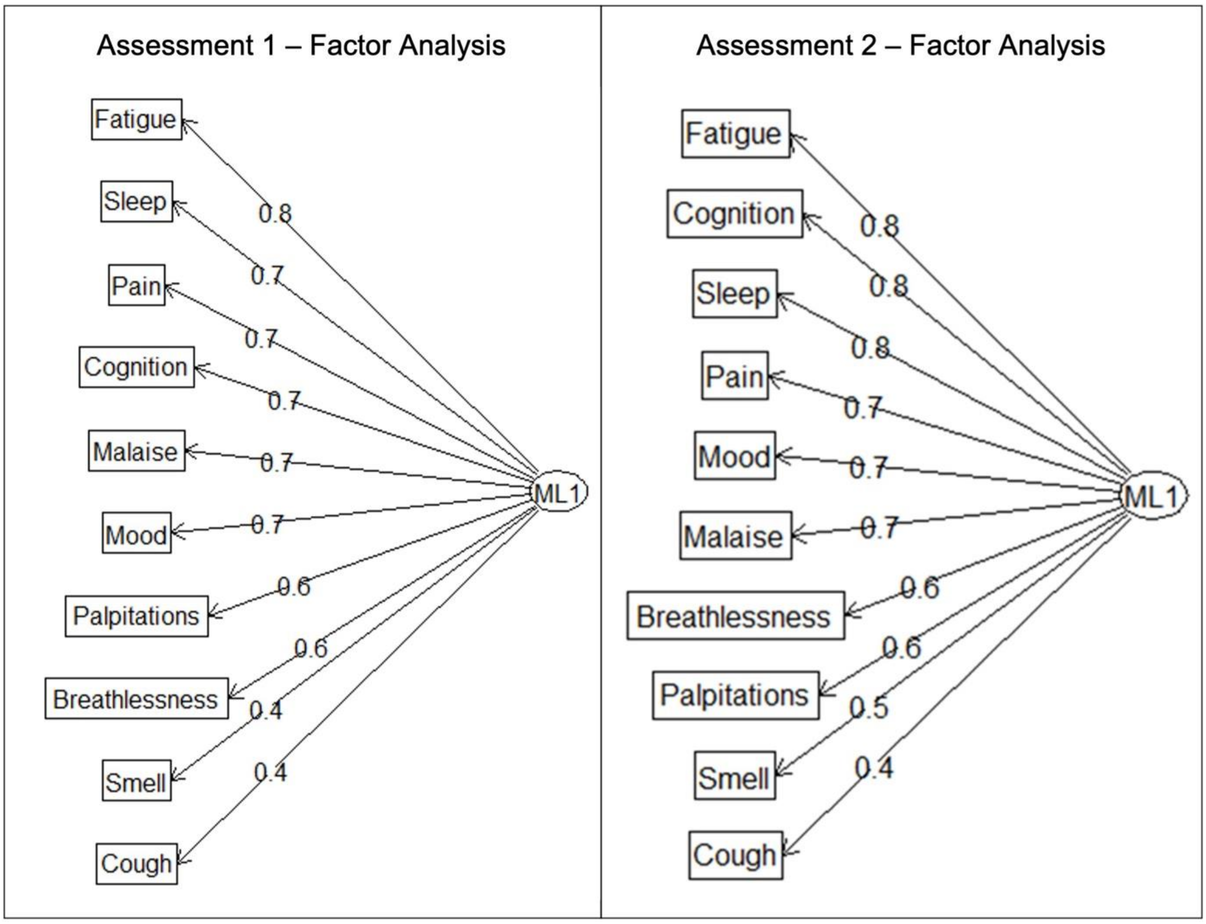

Supplementary Table S2. Polychoric Factor Analysis – Functional Disability – Assessments 1 and 2

**Assessment 1 – Factor loadings**		**Assessment 2 – Factor Loadings**	
Communication	0·54	Communication	0·63
Social	0·75	Social	0·79
ADL	0·84	ADL	0·83
Personal care	0·86	Personal care	0·86
Mobility	0·83	Mobility	0·81
Eigenvalue	2·98	Eigenvalue	3·10
Proportion Variance explained	0·60	Proportion Variance explained	0·62

ADL = Activities of Daily Living

Supplementary Figure S3. Factor Structure – Functional Disability – Assessments 1 and 2

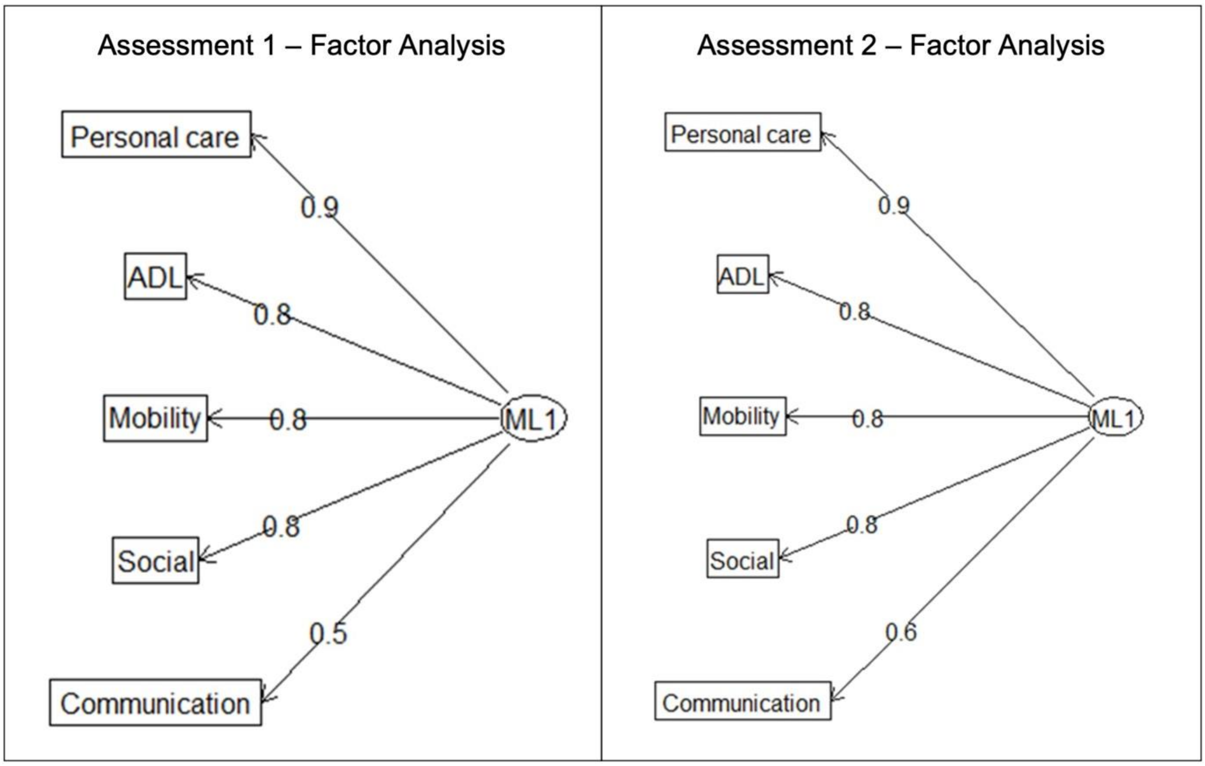

Supplement: Supplementary file 1 [file jcm-13-01908-s001.zip › jcm-2912018-supplementary.pdf]
